# Directed Induction of Functional Motor Neuron-Like Cells from Genetically Engineered Human Mesenchymal Stem Cells

**DOI:** 10.1371/journal.pone.0035244

**Published:** 2012-04-05

**Authors:** Hwan-Woo Park, Jung-Sun Cho, Chul-Kyu Park, Sung Jun Jung, Chang-Hwan Park, Shin-Jae Lee, Seog Bae Oh, Young-Seok Park, Mi-Sook Chang

**Affiliations:** 1 Department of Oral Anatomy, Dental Research Institute and School of Dentistry, Seoul National University, Seoul, Republic of Korea; 2 Department of Physiology, Dental Research Institute and School of Dentistry, Seoul National University, Seoul, Republic of Korea; 3 Department of Physiology, College of Medicine, Hanyang University, Seoul, Republic of Korea; 4 Graduate School of Biomedical Science and Engineering, College of Medicine, Hanyang University, Seoul, Korea; 5 Department of Microbiology, College of Medicine, Hanyang University, Seoul, Korea; 6 Department of Orthodontics, Dental Research Institute and School of Dentistry, Seoul National University, Seoul, Republic of Korea; 7 Neuroscience Research Institute, Seoul National University, Seoul, Republic of Korea; Michigan State University, United States of America

## Abstract

Cell replacement using stem cells is a promising therapeutic approach to treat degenerative motor neuron (MN) disorders, such as amyotrophic lateral sclerosis and spinal cord injury. Human bone marrow-derived mesenchymal stem cells (hMSCs) are a desirable cell source for autologous cell replacement therapy to treat nervous system injury due to their plasticity, low immunogenicity, and a lower risk of tumor formation than embryonic stem cells. However, hMSCs are inefficient with regards to differentiating into MN-like cells. To solve this limitation, we genetically engineered hMSCs to express MN-associated transcription factors, Olig2 and Hb9, and then treat the hMSCs expressing Olig2 and Hb9 with optimal MN induction medium (MNIM). This method of induction led to higher expression (>30% of total cells) of MN markers. Electrophysiological data revealed that the induced hMSCs had the excitable properties of neurons and were able to form functional connections with muscle fibers *in vitro*. Furthermore, when the induced hMSCs were transplanted into an injured organotypic rat spinal cord slice culture, an *ex vivo* model of spinal cord injury, they exhibited characteristics of MNs. The data strongly suggest that induced Olig2/Hb9-expressing hMSCs were clearly reprogrammed and directed toward a MN-like lineage. We propose that methods to induce Olig2 and Hb9, followed by further induction with MNIM have therapeutic potential for autologous cell replacement therapy to treat degenerative MN disorders.

## Introduction

Motor neurons (MNs) are essential effector cells for the control of motor function. Degenerative MN diseases, such as amyotrophic lateral sclerosis (ALS) and spinal muscular atrophy, are devastating disorders associated with loss of MNs [1], [2]. Cell replacement therapy using stem cells is a promising therapeutic option for degenerative MN diseases because MNs located in specific regions of the central nervous system have been linked to these disorders [3], [4]. Recent studies have demonstrated the *in vitro* differentiation of human embryonic stem cells (hESCs) into MNs [5]. However, the use of hESCs in clinical settings causes ethical concerns and is hindered by safety issues, such as teratoma formation and immune rejection [6], [7]. In addition, feeder cells as well as animal fetal calf sera that have been used to culture hESCs pose safety concerns for clinical application [7].

Bone marrow-derived mesenchymal stem cells (MSCs) have self-renewal capacity and are multipotent, with the capability to differentiate into multiple mesodermal cells [8]–[10]. They can also transdifferentiate into neuron-like cells, based on phenotype of various neuronal markers and functional neuronal activity [11]–[14]. Thus, due to their high plasticity and low immunogenicity, human MSCs (hMSCs) could be ideal for autologous cell therapy to treat the injured nervous system, including degenerative MN diseases [15], [16]. In addition, since MSCs do not appear to require major histocompatibility match for transplantation, they could be easily available in a clinical setting, which makes them considered as ‘off-the-shelf stem cells’ for clinical application [17], [18].

The transcriptional pathways that guide motoneuronal specification during vertebral development have been well characterized [19]. For example, transcription factors Olig2 and Hb9 are essential for MN development and differentiation [20], [21]. Basic helix-loop-helix transcription factor Olig2 has been shown to be important in the differentiation of oligodendrocytes and MNs *in vivo*. Olig2 promotes MN development through repressing the expression of antagonists of MN generation, such as Irx3, Scl and other unidentified target genes [22]. Olig2 has a key role in specifying pan-neuronal properties of developing MNs and is selectively expressed by MN progenitors, and precedes the expression of downstream determinants of MN identity, including the homeodomain transcription factors MNR2 and Lim3 [21]. Expression of Olig2 also leads to the expression of post-mitotic MN markers, including Islet-1 and Hb9, homeodomain transcription factors, in neural progenitor cells [21]. Homeodomain transcription factor Hb9 is expressed selectively in embryonic MNs, and plays an essential role in MN development and differentiation [20]. Hb9 triggers cell cycle exit, and an MN marker Islet-1 expression in post-mitotic MN precursors [23].

It was reported that simultaneous expression of Olig2 and Hb9 could drive human adult olfactory neuroepithelial-derived progenitors to differentiate into MNs *in vitro* [24]. Recent studies reported on non-neural human somatic cells converting into neurons by lineage-determining transcription factors, such as Brn2, Ascl1, Myt1l, and NeuroD1 [25]. However, there is a gap in the literature regarding the capability of hMSCs to differentiate into MNs.

Recent studies demonstrated that various key lineage-specific inducing factors have effects on differentiation into specific cell types. The signaling molecule retinoic acid (RA) plays a key role in neurogenesis [26]–[28]. Forskolin (FSK) increases intracellular cAMP, and can promote axonal elongation [29], [30]. Sonic hedgehog (SHH) regulates the generation of MNs and dopaminergic neurons in the ventral region of the embryonic central nervous system (CNS) [31], [32]. RA and SHH are required to regulate the expression of homeodomain and basic helix-loop-helix transcription factors, which regulate the specification of MNs in the ventral spinal cord [27], [33]–[35].

Here, we test the hypothesis that hMSCs can be induced into MN-like cells in a 2-step process. We ectopically expressed Olig2 and Hb9 in hMSCs and then induced with a motor neuron induction medium (MNIM), which we developed in the laboratory. The simultaneous expression of these factors could facilitate the induction of hMSCs into MN-like cells under an optimized MNIM with RA, FSK and SHH. The induced Olig2/Hb9-expressing hMSCs (EOH cells) showed marked expression of MN markers. These induced hMSCs were also able to form functional connections with muscle fibers *in vitro*. Furthermore, they could maintain their neuron-like morphology and neuronal marker expression even at 2 days after transplantation into the spinal cord slice cultures. Our study provides a new fundamental basis for autologous cell replacement therapy to treat degenerative MN diseases.

## Results

### MN Induction of hMSCs

**Figure 1:**
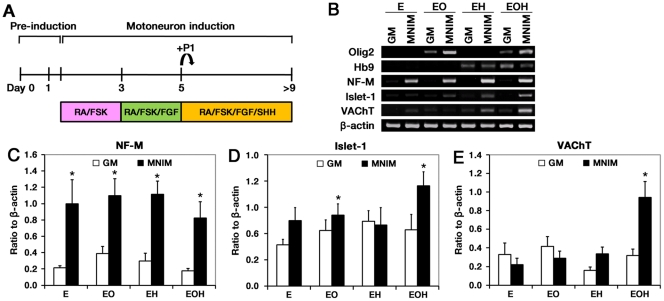
MN marker gene expression in hMSCs expressing Olig2 and Hb9 in the presence of MNIM. (**A**) Schematic illustration of the protocol used for MN induction of hMSCs. (**B**) RT-PCR analyses of hMSCs expressing EGFP (E), EGFP-Olig2 (EO), EGFP-Hb9 (EH), and EGFP-Olig2-Hb9 (EOH) with MNIM treatment were preformed. Quantification of NF-M (**C**), Islet-1 (**D**), and VAChT (**E**) PCR products. The mRNA level of a given gene was quantified by densitometry and normalized to the corresponding β-actin level. The bars represent mean±SEM of at least three independent experiments. *P<0.05 versus uninduced E. The significance was determined by Mann-Whitney test with Fisher’s LSD *post hoc* test.

A previous study has reported that simultaneous expression of Olig2 and Hb9 was sufficient to induce human olfactory neuroepithelial progenitors to MN-like cells *in vitro* [24]. However, as far as we are aware, there is no report that describes the induction of hMSCs into MN-like cells. Thus, in this study, we sought to develop an efficient protocol to generate MN-like cells is partly depicted in Figure 1A.

We used a 2-step process to differentiate hMSCs into MN-like cells. First, we ectopically expressed Olig2 and/or Hb9 in hMSCs using EGFP-Olig2-Hb9 (EOH), EGFP-Olig2 (EO) and EGFP-Hb9 (EH)-containing viral vectors. Control hMSCs were infected with a virus containing vector alone (E). The transfectants were then compared for MN differentiation. We determined whether the expression of Olig2 and/or Hb9 induced the expression of neuronal/motoneuronal marker genes in growth medium alone (GM). We performed RT-PCR for Olig2, Hb9, neurofilament-M (NF-M), Islet-1, and vesicular acetylcholine transporter (VAChT). Olig2 mRNA was detected only in EO cells and EOH cells. Likewise, Hb9 mRNA was detected only in EH cells and EOH cells (Fig. 1B). However, there was no significant difference in the mRNA levels of NF-M, Islet-1, and VAChT between the groups (Fig. 1B), indicating that the expression of Olig2 and Hb9 were not sufficient to induce the hMSCs to MN cells.

We next asked if MNIM treatment of the transfectants above could differentiate the cells to MN cells. The cells were treated with MNIM using the scheme shown in Figure 1A. The MNIM protocol for hMSCs was developed in our laboratory based on the previous studies of inducing hESCs to MN-like cells [5].

We also performed RT-PCR for Olig2, Hb9, NF-M, Islet-1, and VAChT in MNIM-treated cells (Fig. 1B). Interestingly, MNIM treatment significantly increased NF-M mRNA levels in all groups (*P*<0.05) when compared with untreated cells (E) (Fig. 1B, C). These results suggest that MNIM treatment is sufficient to induce the expression of the neuronal marker gene, *NF-M*, in hMSCs. However, the mRNA for a MN marker, Islet-1, was only significantly increased when MNIM was added to EO cells and EOH cells (*P*<0.05) (Fig. 1B, D). Furthermore, we observed a significant increase in the expression of another MN marker, VAChT, only in MNIM-treated EOH cells (*P*<0.05) (Fig. 1B, E). Although there was an increase in VAChT mRNA expression in MNIM-treated EH cells, this increase did not reach statistical significance (Fig. 1B, E).

In summary, NF-M, Islet-1, and VAChT mRNA levels were increased significantly only in MNIM-treated EOH cells as compared to untreated EOH cells. These results suggest that both 2-steps, including coexpression of Olig2 and Hb9 and MNIM treatment, are required to induce hMSCs to MN-like cells.

### Characterization of hMSCs Induced to MNs

**Figure 2:**
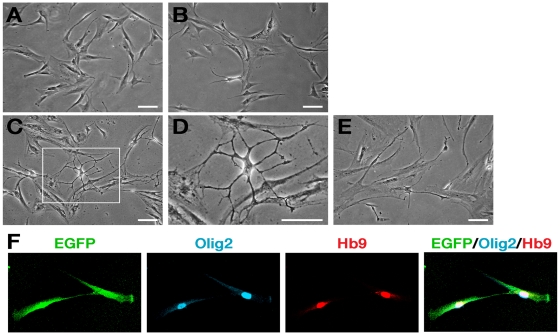
Changes in cell morphology of hMSCs expressing Olig2-Hb9 after the induction. Bright-field images of uninduced EOH cells (**A**), EOH cells induced for 5 days (**B**), completely induced EOH cells (**C**), and induced EOH cells further cultured in growth medium for 2 days after complete induction (**E**). (**D**) A higher magnification of the corresponding box shown in (**C**). (**F**) GFP (green), Olig2 (blue) and Hb9 (red) were expressed in EOH cells. Scale bar: 100 µm.

Next, we further characterized the genetically engineered hMSCs induced to MN-like cells (MNIM-EOH cells). First, we examined whether there were morphological changes during induction with MNIM. Untreated EOH cells exhibited a flattened and symmetrical fibroblast-like morphology (Fig. 2A). However, following exposure to MNIM, EOH cells underwent morphological changes and adopted a neuron-like morphology (structures of cell bodies with long thin processes) (Fig. 2B–D). Morphological changes were apparent in ∼7% of cells at day 5 post-induction (Fig. 2B) and in >32% of cells at day 9 post-induction (Fig. 2C, D, Table 1).

**Table 1:** Quantitative analysis of cell morphology (% of total cells).

	MSC-like morphology	Neuronal-like morphology
Untreated EOH-hMSC	100	0
Day 3 post-induction	98.8±0.7	1.2±0.7
Day 5 post-induction	92.9±2.2	7.1±2.2
Induced EOH-hMSC	67.1±2.5	32.9±2.5
Induced EOH-hMSC+GM	82.2±2.7	17.8±2.7

The values are expressed as the mean±SEM.

To determine whether the morphology of induced EOH cells could be maintained without the inducing factors, we further cultured fully induced cells in growth medium only. It has been reported that cell-doubling time of hMSCs is 48 to 72 hours [36]. We also calculated the doubling time of uninduced EOH cells (49.87 hours, Figure S1). Thus, we hypothesized that if cells were not induced to neurons, they would undergo actin reorganization for cell division during 2 days in growth medium instead of maintaining neuron-like morphology. However, approximately 18% of the induced EOH cells still maintained neuron-like morphology even after 2 days without MNIM (Fig. 2E, Table 1). Thus, this morphological change was not due to actin reorganization by the inducing factors since these cells could maintain changed morphology for 2 days even without MNIM. We also confirmed that Olig2 and Hb9 were still expressed in these cells by immunocytochemistry as in uninduced EOH cells (Fig. 2F).

**Figure 3:**
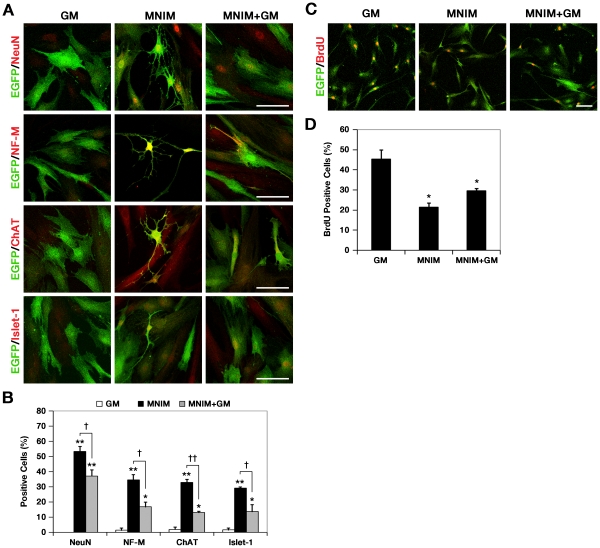
Increased expression of mature neuronal/motoneuronal markers and cell-cycle arrest occur in MNIM-treated EOH **cells.** (**A**) Immunocytochemistry analysis of neuronal and MN-specific markers in uninduced EOH cells, MNIM-EOH cells, and MNIM-EOH cells further cultured in growth medium for 2 days after complete induction. Each row represents NeuN (red), NF-M (red), ChAT (red), and Islet-1 (red) staining with EGFP (green). Scale bar: 50 µm. (**B**) Quantitative analysis of NeuN, NF-M, ChAT, and Islet-1 expression. Fifty to 150 cells per group were analyzed in randomly chosen fields. The bars represent mean±SEM of at least three independent experiments. * *P*<0.05, ** *P*<0.001 versus GM. † *P*<0.05, †† *P*<0.001 versus MNIM. The significance was determined by ANOVA followed by Fisher’s LSD *post hoc* test. (**C**) Immunocytochemistry of BrdU incorporation (red) and EGFP (green) in EOH cells cultured in GM, MNIM, and MNIM+GM. (**D**) Quantitative analysis of BrdU incorporation. Fifty to 150 cells per group were analyzed in randomly chosen fields. The bars represent mean±SEM of at least three independent experiments. * *P*<0.05 versus GM. The significance was determined by ANOVA followed by Fisher’s LSD *post hoc* test.

We further characterized the cells by determining for the expression of mature neuronal and MN-specific marker proteins, neuronal nuclei (NeuN), NF-M, choline acetyltransferase (ChAT), and Islet-1 in MNIM-EOH cells (Fig. 3A). Immunocytochemistry analyses indicated that the percentage of NeuN, NF-M, ChAT, or Islet-1 protein expression was significantly increased in MNIM-EOH cells (*P*<0.001) when compared with uninduced EOH transfectants (GM), which expressed low to undetectable levels of these markers (Fig. 3B). In induced EOH cells further cultured in growth medium for 2 days after 9-day complete induction (MNIM+GM), the percentages of NeuN, NF-M, ChAT, or Islet-1-positive cells were much higher (>13% of total cells) than in EOH cells cultured in growth medium (GM), although, significantly lower than MNIM-EOH cells (Fig. 3B). Taken together, these results indicate that EOH cells exposed to MNIM adopted morphological and molecular markers, consistent for MNs.

### Cell Cycle Arrest Occurs in hMSCs Induced to MNs

Previously, we demonstrated that Schwann cell induction of hMSCs was associated with cell-cycle arrest [37]. Thus, we examined whether MN induction of hMSCs could also lead to cell cycle arrest. This question was addressed in studies to monitor bromodeoxyuridine (BrdU) incorporation (Fig. 3C). The number of BrdU-incorporated cells was significantly decreased in MNIM (21.4%±2.1%) and MNIM+GM (29.5%±1.1%) compared to GM (45.4%±4.4%) (*P*<0.05) (Fig. 3D). These results indicate that the induction of hMSCs to MN correlated with reduced cell cycling.

### Excitable Properties of MNIM-EOH Cells

**Figure 4:**
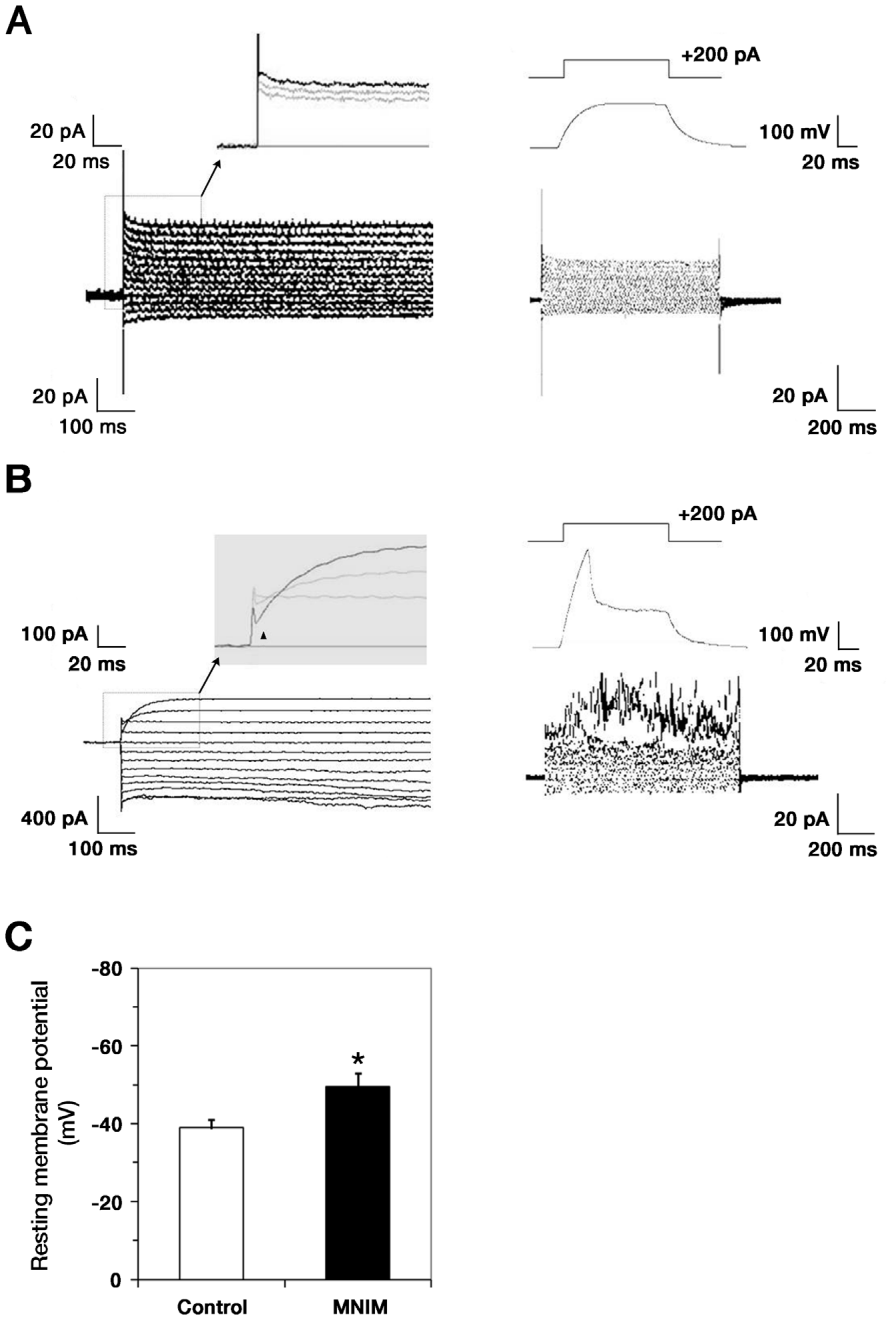
Excitable properties of MNIM-treated EOH cells. Representative perforated patch clamp recordings from uninduced EOH cells (**A**) or MNIM-EOH cells (**B**). The cells were held at −60 mV and currents elicited by stepping from −90 to +30 mV in 10 mV steps. MNIM-EOH cells express robust outward K+ currents but very small inward Na+ currents (filled upright triangle). Current injection (200 pA, 100 ms) induced action potential in MNIM-EOH cells, not in uninduced EOH cells. (**C**) Resting membrane potential in uninduced EOH cells and MNIM-EOH cells. The bars represent mean ± SEM. * *P*<0.05. The significance was determined by Student’s *t* test.

Neuronal properties of MNIM-treated EOH cells were studied with perforated patch clamp recording in the voltage clamp mode using a step protocol from −90 mV to +30 mV. This protocol failed to elicit Na+ currents in EOH cells (Fig. 4A; n = 10/10), but elicited small inward Na+ currents in more than a half of MNIM-EOH cells (Fig. 4B, triangles; 8 out of 11 cells). These results suggest that although MNIM-EOH cells express functional K+ channels, they express low levels of voltage-gated Na+ channels. To further assess the excitable properties of MNIM-EOH cells, we performed perforated patch clamp recording in the current clamp mode. Resting membrane potential of MNIM-EOH cells (n = 11) ranged from −30 mV to −64 mV, and more than a half of these cells exhibited evoked action potentials (n = 8/11). In contrast, the membrane potential of EOH cells (n = 10/10) ranged from −29 mV to −48 mV, and none of the cells exhibited evoked action potentials (Fig. 4C). These results are consistent with their inability to fire spontaneous or evoked action potentials. Taken together, the results indicate that MNIM-EOH cells have the excitable properties of neurons.

### MNIM-EOH Cells can Induce AChR Clustering and Form Functional Connections with Myotubes

**Figure 5:**
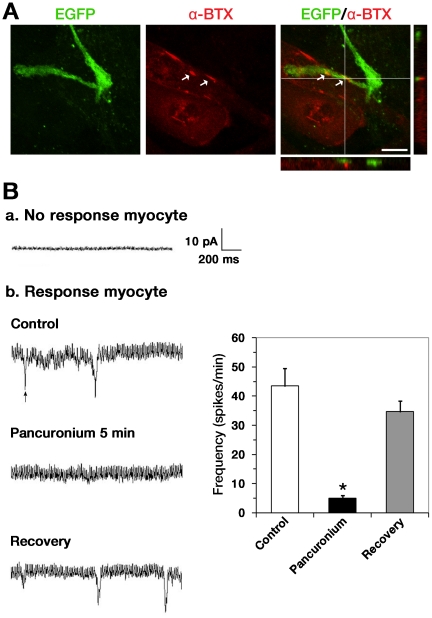
MNIM-EOH cells can trigger AChR clustering and form functional connections with cocultured myotubes. (**A**) Fluorescence images of MNIM-EOH cells at 1 day after cocultured with C2C12 myotubes. α-BTX staining revealed AChR clustering on C2C12 muscle fibers. Confocal Z-stack imaging, in a line through the region of apparent colocalization, confirmed EGFP+ axons in close proximity to AChRs (arrows). Scale bars: 10 µm. (**B**) End-plate currents (EPCs) were recorded from myotubes located in close proximity to MNIM-EOH cells. EPCs were blocked from the same cell after application of 15 µM pancuronium. However, after washing out of pancuronium, EPCs could be recorded again. The bars represent mean±SEM. * *P*<0.05. The significance was determined by Student’s *t* test.

The primary function of MNs is to trigger muscle contraction by releasing Ach at the neuromuscular junction (NMJ). NMJs contain acetylcholine receptor (AChR) clustering (reviewed in [38]). To examine whether MNIM-EOH cells could form NMJs with cocultured C2C12 myotubes, we determined if there is colocalized EGFP+ axons with rhodamine conjugated α-bungarotoxin (α-BTX), which labels clustered AChRs. Indeed, AChR clustering occurred on myotubes immediately adjacent to the extended EGFP+ axons (Fig. 5A, arrow).

In order to determine whether MNIM-EOH cells can establish functional connections with myotubes, we performed whole-cell recordings from the individual myotubes. Four end-plate currents (EPCs) were recorded from individual myotubes 9 days after plating (Fig. 5B; a single EPC is indicated with an arrowhead). EPCs were completely blocked from the same cell shortly after bath application of pancuronium (15 µM for 5 min; n = 4, Fig. 5B), indicating that the EPCs were generated due to cholinergic transmission. The data were supported by the full recovery of EPCs after washing out of pancuronium (n = 4, Fig. 5B). Therefore, these results indicate that MNIM-EOH cells can induce AChR clustering and form functional NMJs with myotubes *in vitro*.

### *Ex Vivo* Assessment of Transplanted MNIM-EOH Cells in Demyelinated Organotypic Spinal Cord Slice Culture

**Figure 6:**
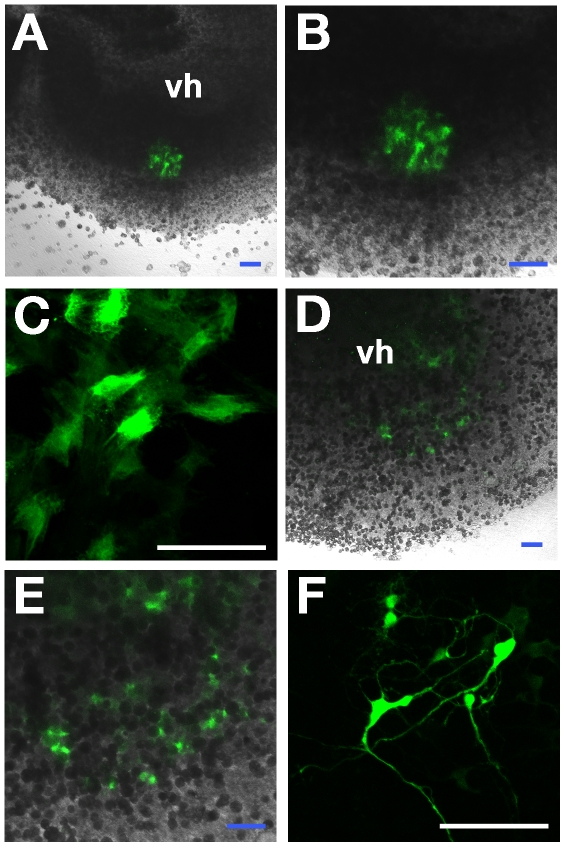
*Ex vivo* assessment of EOH cells or MNIM-EOH cells after transplantation into the spinal cord slice culture. Confocal images of uninduced EOH cells (green) (**A-C**) or MNIM-EOH cells (green) (**D-F**) at 2 days after transplantation into the ventral horn area (vh) of a spinal cord slice. (**B**) and (**E**) are a higher magnification of (**A**) and (**D**), respectively. (**C**) Confocal image of transplanted EOH cells with a flattened and symmetrical fibroblast-like morphology. (**F**) Confocal image of transplanted MNIM-EOH cells with a neuron-like morphology. Scale bars: 100 µm.

The *ex vivo* properties of MNIM-EOH cells were studied after transplantation into the lysolecithin-treated rat spinal cord slice culture. Organotypic spinal cord slice cultures were prepared as previously described by our group [37], [39]. Although a number of cells were washed away during the transplantation procedure, many EGFP+ cells were found in the ventral region of the spinal cord slices when examined even 2 days later (Fig. 6). Untreated EOH cells maintained a flattened and symmetrical fibroblast-like morphology in slices (Fig. 6A-C). However, MNIM-EOH cells could maintain their differentiated neuron-like morphology in slices (Fig. 6D-F). Furthermore, they seemed to be connected with adjacent neurons, although it is not clear whether they had functional synapses.

**Figure 7:**
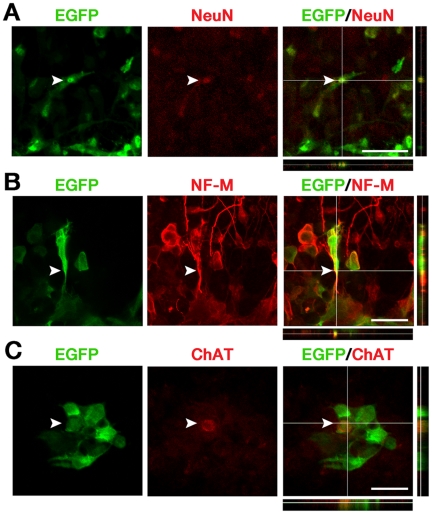
MNIM-EOH cells express mature neuronal/MN markers after transplanted into the injured spinal cord slice culture. Confocal microscopy analysis of MNIM-EOH cells (green) immunostained with anti-NeuN (red) (**A**), anti-NF-M (red) (**B**), and anti-ChAT (red) (**C**) antibodies. In the slices, 37 of 472 EGFP+ cells were NeuN+, 20 of 316 EGFP+ cells were NF-M+, and 14 of 469 EGFP+ cells were ChAT+. Colocalization of EGFP and NeuN, NF-M, or ChAT in a single cell was confirmed by z-axis stack analysis. At least 4 slices were analyzed. Scale bars: 50 µm.

We further investigated whether these transplanted MNIM-EOH cells expressed mature neuronal and MN-specific marker proteins. Immunohistochemical analysis indicated that < 8% of all EGFP+ cells expressed a neuronal marker NeuN (Fig. 7A), and <7% of all EGFP+ cells expressed NF-M (Fig. 7B) at 2µdays after transplantation. Furthermore, <3% of all EGFP+ cells expressed an MN-specific marker ChAT (Fig. 7C). These results suggest that MNIM-EOH cells can survive in the injured spinal cord slice while maintaining expression of neuronal and MN-specific markers.

## Discussion

ESCs, by in vitro methods, can differentiate into functional MNs when treated with SHH and RA [5], [35], [40]. However, the potential for immune rejection and the possibility of teratoma formation may limit clinical application. Our study demonstrates that hMSCs can be induced into MN-like cells through genetic engineering. Combined induction of Olig2 and Hb9, and exposure to SHH and RA induced the generation of MN-like cells *in vitro*. The efficiency of induction was 29–33% based on MN-specific marker and morphological analyses. In addition, approximately 18% of the induced EOH cells still maintained neuron-like morphology even after 2 days without MNIM, indicating that the morphological changes occurred during induction with MNIM could be maintained in these cells. We previously showed that the Schwann cell induction of hMSCs led to cell cycle arrest during differentiation [37]. The current study also demonstrated that cell cycle arrest occurred during the MN induction.

The combination of RA and SHH stimulates the expression of transcription factors which play important roles in neuronal differentiation [27], [33], [34]. However, morphogens, including RA and SHH, are not sufficient to induce MN differentiation of neural stem cells or neural precursors [24], [41], [42]. Likewise, in this study, RT-PCR analyses also demonstrated that SHH and RA had only minimal effects on MN induction of hMSCs. For example, MNIM-treated hMSCs without genetic modification, expressed low mRNA levels of Islet-1 or VAChT. We also observed that only a few cells exhibited a neuron-like morphology (data are not shown). This suggests that the missing transcription factors are required to drive *in vitro* MN differentiation of hMSCs because the response of hMSCs to RA and SHH was incomplete.

Previously, it was reported that simultaneous transfection of human olfactory neuroepithelial-derived progenitors with Olig2 and Hb9 combined with other factors lead to the expression of MN lineage-restricted markers [24]. Genetically engineered fetal rat neural precursors with transcription factors HB9, Nkx6.1, and Neurogenin2 can also induce the generation of MNs *in vitro* in the presence of RA and SHH [41]. In this study, we developed the method to induce to MN-like cells from genetically engineered hMSCs instead of neural progenitors or precursors, since we believe that hMSCs have more therapeutic potential for autologous cell replacement therapy to treat degenerative MN disorders.

In this study, hMSCs ectopically expressing Olig2 and Hb9 have a significantly increased MN-specific marker expression and the capacity to induce AChR clustering on myotubes after being treated with MNIM, containing inducing factors. Consistent with the roles of inducing factors that stimulate neurogenesis, simultaneous expression of Olig2 and Hb9 cannot increase mRNA levels of neuronal/MN markers without MNIM treatment. These data suggest that genetic modification of hMSCs to express Olig2 and Hb9 renders them capable of inducing MN differentiation only in the presence of MNIM.

Our study demonstrates that hMSCs can be induced into functional MN-like cells after a 2-step process, including ectopic expression of transcription factors and MNIM treatment. MNIM-EOH cells have an increased expression of mature neuronal and MN-specific markers, including NeuN, NF-M, ChAT, and Islet-1. MNIM-EOH cells can also form functional connections with C2C12 myotubes. Based on the electrophysiological properties of the induced EOH cells, our data demonstrate that MNIM-EOH cells are clearly reprogrammed and directed toward a MN-like lineage. For example, although Na+ current recorded in MNIM-EOH cells is small, its amplitude can be estimated at ∼300 pA in voltage of −10 mV. This 300 pA inward current is thought to be enough to generate the action potential, although Na+ current of MNIM-EOH cells is smaller than Na+ current of neurons. In current clamp mode, 200 pA current injection can induce the depolarization and single action potential. Thus, small Na+ current of MNIM-EOH cells can induce the action potential-like excitability, not the fast action potential of neuron.

We also demonstrate that MNIM-EOH cells can survive and maintain neuron-like phenotype even at 2 days after transplantation into the injured spinal cord slice. One of the major hurdles with neuronal replacement therapy is the integration of transplanted neurons into the existing neural networks. Therefore, further studies are needed to confirm whether these survived cells can be possibly integrated into pre-existing neural networks in the injured spinal cord slice while maintaining expression of neuronal and MN-specific markers. It will also be interesting to investigate whether these cells can innervate appropriate peripheral targets to replace missing or degenerated MNs in the animal model of MN diseases, such as ALS.

Although we ectopically expressed Olig2 and Hb9 in hMSCs, the transdifferentiation potential seems to depend on each subpopulation of hMSCs. This may explain why it still remains controversial whether MSCs can transdifferentiate into functional neurons [11], [12], [43], [44]. Several recent studies have reported that a subpopulation of MSCs in bone marrow may originate from neural crest stem cells (NCSCs) during development [45], [46]. NCSCs are known to have the potential to differentiate into neurons, glial cells, and myofibroblasts [47], [48]. Thus, it has been suggested that transdifferentiation potential into neurons from MSCs may reflect the presence of NCSCs of the bone marrow [45], [46]. In our previous study, we also observed that several neural crest cell markers were highly expressed in control hMSCs and that p75NTR mRNA levels were highly induced in response to serum [37]. Further studies are needed to determine whether these molecular changes occurred specifically within a subpopulation of hMSCs with a neural crest origin. Therefore, identification of this subpopulation with high capacity to differentiate into neurons will enhance the potential of hMSC administration as a therapeutic strategy to treat degenerative MN diseases.

In summary, we demonstrate that hMSCs can be induced into functional MN-like cells in a 2-step process, including ectopic expression of transcription factors and an optimized induction protocol. The induced cells have marked expression of MN markers and the excitable properties of neurons. They can form functional connections with muscle fibers *in vitro* and maintain their neuron-like morphology and neuronal marker expression even at 2 days after transplantation into the injured spinal cord slice. Neuronal replacement of missing or degenerated neurons will likely enhance recovery from degenerative MN diseases. Therefore, our study provides and tests the potential of hMSC administration as a therapeutic strategy to treat degenerative MN diseases.

## Materials and Methods

### Human Mesenchymal Stem Cell Culture

Cryopreserved adult hMSCs (Poietics® normal human mesenchymal stem cells) were purchased from Cambrex (Walkersville, MD). hMSCs (passages 4 through 12) were cultured in Dulbecco’s modified Eagle’s medium (DMEM)-low glucose (Hyclone, Logan, UT) containing 10% fetal bovine serum (FBS; Gibco-BRL, Carlsbad, CA) at 37°C with 5% CO_2_.

### Expression Vector Construction and Infection

A full-length mouse Olig2 cDNA fragment (GenBank Accession number: NM_016967) was constructed by inserting the *EcoR*I*-EcoR*V digested gene fragment into an *EcoR*I*-Pme*I-digested lentiviral vector, pgk EGFP-mCMV-IRESpuro (pLentiM1.41; Macrogen Inc., Seoul, Korea) (EO). The full-length mouse Hb9 cDNA (GenBank Accession number: NM_019944) was treated with T4 polymerase to produce blunt ends, and the blunt-ended Hb9 cDNA was inserted into the pLentiM1.41 (EH). For the coexpression of Olig2 and Hb9, Olig2 and Hb9 cDNA fragments were cloned into the multicloning sites of the retroviral vector plasmid EGFPBsd-CLCBC4 (EOH). The construct was confirmed through multiple and combined digestions, and the nucleotide sequence of the construct was verified by DNA sequencing. Viral particles were produced by transfecting the retrovirus packaging cell line 293T with each plasmid using Lipofectamine (Invitrogen, Carlsbad, CA), and supernatants containing viral particles were harvested 72 hours after incubation. For viral transduction, hMSCs were incubated for 6 hours with the viral supernatant (3–5×10^6^ particles/ml) containing polybrene (1 µg/ml, Sigma-Aldrich, St. Louis, MO), followed by a medium change.

### MN Induction of Human Mesenchymal Stem Cells

Subconfluent hMSCs were incubated in growth medium with 1 mM β-mercaptoethanol (Sigma-Aldrich) for 24 hours and were subsequently treated with 2 mM β-mercaptoethanol. After 3 hours, cultures were transferred to growth medium containing 1 µM retinoic acid (RA; Sigma-Aldrich) and 5 µM forskolin (FSK; Sigma-Aldrich). At day 3, the cultures were maintained in growth medium supplemented with 1 µM RA, 5 µM FSK and 10 ng/ml recombinant human basic-fibroblast growth factor (bFGF; Peprotech, Rocky Hill, NJ). After an additional 4–6 days of culture, with media changes every 2 days, the cells were induced in the presence of 1 µM RA, 5 µM FSK, 10 ng/ml bFGF and 200 ng/ml recombinant human sonic hedgehog (SHH; R&D Systems, Hornby, Ontario, Canada).

### Reverse Transcription-polymerase Chain Reaction

RNA was extracted using the TRIzol reagent (Invitrogen), followed by DNase treatment and then reverse transcription to cDNA using M-MLV reverse transcriptase (Invitrogen). Aliquots of cDNA (200 ng) were used as templates in the PCR reactions (50 µl), containing 200 nM dNTPs, 100 pM of each primer pair, and 0.5 U of Taq DNA polymerase (Takara Bio, Tokyo). Table 2 displays the primer probe sets used for the PCR reactions. Products were visualized by electrophoresis using 2% agarose gel. Normalization was performed through a semiquantitative analysis of transcript abundance, based on β-actin expression.

**Table 2:** Polymerase chain reaction primer pairs.

Gene	Accession number	Primer sequence	Predicted size (base pairs)	Anneal (°C)	Cycle no.
Olig2	NM_005806	5′-aaggaggcagtggcttcaagtc-3′	315	60	35
		5′-cgctcaccagtcgcttcatc -3′			
Hb9	NG_013212	5′-gcaggcggcgctctac-3′	258	56	35
		5′-ttccccaggaggttcgac-3′			
NF-M	BC096757	5′-tgggaaatggctcgtcatttg-3′	333	57	35
		5′-cttcatggaaacggccaattc-3′			
Islet-1	NM_002202	5′-gcagcatcggcttcagcaag-3′	356	60	35
		5′-gtagcaggtccgcaaggtg-3′			
VAChT	U10554	5′-tggcgctgttactggcaac-3′	249	60	35
		5′-tcttcacgtcttcgctctc-3′			
β-actin	NM_001101	5′-ccacgaaactaccttcaactcc-3′	270	55	30
		5′-tcatactcctgctgcttgctgatcc-3′			

### Immunofluorescence

Cells were fixed with 4% paraformaldehyde, permeabilized and blocked with 0.2% Triton X-100 and 3% bovine serum albumin (BSA) in phosphate-buffered saline (PBS). Cells were incubated overnight at 4°C with the following primary antibodies: mouse monoclonal anti-NeuN, 1∶500 (Chemicon, Temecula, CA), rabbit polyclonal anti-neurofilament-M (NF-M), 1∶500 (Chemicon), mouse monoclonal anti-choline acetyltransferase (ChAT), 1∶200 (Chemicon), and mouse monoclonal anti-Islet-1, 1∶10 (Developmental Studies Hybridoma Bank [DSBH], Iowa City), mouse monoclonal anti-Hb9, 1∶50 (DSBH), and rabbit polyclonal anti-Olig2, 1∶5,000 (kindly provided by Dr. Charles D. Stiles, Harvard Medical School, Boston, MA). After washing with PBS, cells were incubated with the following secondary antibodies: Alexa Fluor 546 anti-mouse IgG (Invitrogen), Alexa Fluor 546 anti-rabbit IgG (Invitrogen), and anti-rabbit Cy5 (Jackson Laboratory, West Grove, PA). Cells were coverslipped with mounting medium (Dako, Glostrup, Denmark), and immunoreactive cells were analyzed under a laser-scanning confocal microscope (Olympus, Tokyo).

### Bromodeoxyuridine Incorporation

The hMSCs were incubated with 10 μM bromodeoxyuridine (BrdU; Sigma-Aldrich) for 24 hours and fixed with 4% paraformaldehyde. The DNA was then denatured with 2 N HCl for 30 minutes at 37°C, and the reaction was neutralized with 0.1 M sodium borate (pH 8.5) for 10 minutes at room temperature. The rest of the protocol was a standard immunocytochemistry. Mouse monoclonal anti-BrdU, 1∶200 (Chemicon) was used as the primary antibody.

### Myotube Co-culture

Undifferentiated mouse myoblast C2C12 cells (American Type Culture Collection, Manassas, VA) were maintained in DMEM with 10% FBS, penicillin and streptomycin. The C2C12 cells were plated onto glass coverslips in six-well plates (2×10^5^ cells/35-mm well) in maintaining medium. After 2 days, C2C12 cells were differentiated in DMEM with 2% horse serum (Gibco-BRL), penicillin and streptomycin for 3 additional days. Differentiated C2C12 myotubes were co-cultured with MNIM-EOH cells for 24 hours. Immunocytochemistry were performed with rhodamine-conjugated α-bungarotoxin, 1∶1000 (α-BTX, Molecular Probes, Eugene, OR). Optical sections (1.0-µm) were collected as stacked z-dimension images, which were inspected orthogonally in both the horizontal and vertical planes.

### Electrophysiological Recordings

MNIM-EOH cells were studied using the perforated patch clamp technique. Patch pipettes were pulled from borosilicate capillaries (PP-83 puller, Narishige, Tokyo, Japan) with resistances of 4–5 MΩ when filled with pipette solution (in mM: 126 K-gluconate, 10 NaCl, 1 MgCl_2_, 0.5 EGTA, 2 NaATP, 0.1 MgGTP. pH adjusted to 7.3 with KOH). In all experiments, the external solution consisted of 145 mM NaCl, 5 mM KCl, 2 mM CaCl_2_, 1 mM MgCl_2_, 10 mM HEPES pH 7.4, and 10 mM glucose. The membrane currents were recorded in the nystatin-perforated patch configuration using an EPC-9 amplifier and Pulse 8.30 software (both from HEKA, Lamprecht, Germany). Nystatin (Sigma-Aldrich) was employed as the permeable agent in the perforated patch-clamped cells to form voltage-insensitive ion pores in the membrane patch that are somewhat selective for cations over anions but are impermeable to multivalent ions or molecules >0.8 nm in diameter. Nystatin was dissolved in dimethyl sulfoxide (DMSO) at 50 mg/ml and then added to the internal solution to yield a final nystatin concentration of 200 µg/ml. The series resistances in perforated patch-clamping neurons were within 30–45 MΩ. The cells were held at −60 mV and currents were elicited by stepping from −90 to +30 mV in 10 mV steps. The recording chamber was gravity superfused with external solution at ∼10 ml/min. The data were digitized at a sampling rate of 3 kHz and were filtered at 10 kHz. Data files were analyzed using Origin 4.1.

### Whole-cell Recordings of Muscle Fibers

Single myotubes near MNIM-EOH cells were visually identified using a fixed-stage microscope (BX50WI, Olympus, Japan) with Nomarski optics and a 40× water-immersion objective. Myotubes with stable resting membrane potentials (Vm) between -50 and −60 mV were included in the analysis. Postsynaptic end-plate currents (EPCs) were amplified via an EPC-9 amplifier and Pulse 8.30 software. Signals were analyzed off-line using the Mini Analysis Program (Synaptosoft, Decatur, GA, http://www.synaptosoft.com).

### Organotypic Spinal Cord Slice Culture

**Ethics statement.** This study was carried out in strict accordance with the recommendations in the Guide for the Care and Use of Laboratory Animals of the National Institutes of Health. The protocol was approved by the Institutional Animal Care and Use Committee of Seoul National University (Permit Number: SNU-091120-2). All surgery was performed under avertin anesthesia, and all efforts were made to minimize suffering.

Organotypic slice cultures were prepared as previously described [37], [39]. Sixteen-day-old Sprague-Dawley rats were anaesthetized with avertin, and their lumbar spinal cords were collected under sterile conditions. Nerve roots and excess connective tissue were removed in cold Hank’s balanced salt solution (HBSS; Gibco-BRL) containing 6.4 mg/ml glucose. The spinal cords were cut into 400-µm slices with a McIlwain tissue chopper (Mickle Laboratory Engineering, Gomshall, Surrey, UK). Four slices were carefully placed on a membrane insert (Millicell-CM; Millipore, Billerica, MA) and placed into a 6-well plate with 1 ml of culture media consisting of 50% Eagle’s minimum essential medium (Gibco-BRL), 25% HBSS, 25% horse serum, 6.4 mg/ml glucose and 20 mM HEPES (Sigma-Aldrich). Slices were cultured at 37°C with 5% CO_2_, and the media were changed twice per week.

### hMSC Transplantation into the Lysolecithin-treated Spinal Cord Slice Culture

At 7 days in vitro (DIV), slices were treated with 0.5 mg/ml lysophosphatidylcholine (lysolecithin; Sigma-Aldrich) for 17 hours at 37°C as previously described [37]. For transplantation, EOH cells or MNIM-EOH cells were trypsinized and centrifuged at 2,000 g for 3 minutes. The pellet was resuspended in PBS, and cells (6×10^3^ cells/1 µl) were transplanted directly into the area of the ventral horn (VH) of each spinal cord slice using aspirator tube assembly for microcapillary pipette (Sigma-Aldrich). After 2-6 days in culture, cells on the slices were analyzed by confocal microscopy after processed for immunofluorescence.

### Statistical Analysis

Data were analyzed using Student’s t test or one-way analysis of variance (ANOVA) followed by post hoc comparisons with Fisher’s protected least significant difference (LSD) test. RT-PCR results were analyzed using the Kruskal-Wallis and Mann-Whitney tests. The data were evaluated using the language R (R Development Core Team 2010) and were expressed as the mean±standard error of mean (SEM). Probability (P) values <0.05 were considered statistically significant.

## Supporting Information

Figure S1 **Doubling time of uninduced EOH cells.** We employed regression with “exponential rise to maximum” type, as y = y_o_ + b(1 – e^–ax^), where y_o_ is the intercept, a is the slope between x and y, and b is the maximum y value. According to the estimations in 99% confidence (*P* < 0.01), N_cell_ at t = 0 corresponds to 2128 and the growth rate (i.e., number of doublings that occur per unit of time) results in 0.0139. Note that our data can be well fitted as N_cell_ = 2128+6469 (1 – e^−0.0139×time^). The doubling time of EOH cells is calculated as ln2/0.0139 = 49.87 hr.
(TIF)
