# Supplementary figures and images for: Directed Induction of Functional Motor Neuron-Like Cells from Genetically Engineered Human Mesenchymal Stem Cells
Source: PLoS One. 2012 Apr 5;7(4):e35244. doi: 10.1371/journal.pone.0035244 (PMC3320649; doi:10.1371/journal.pone.0035244)

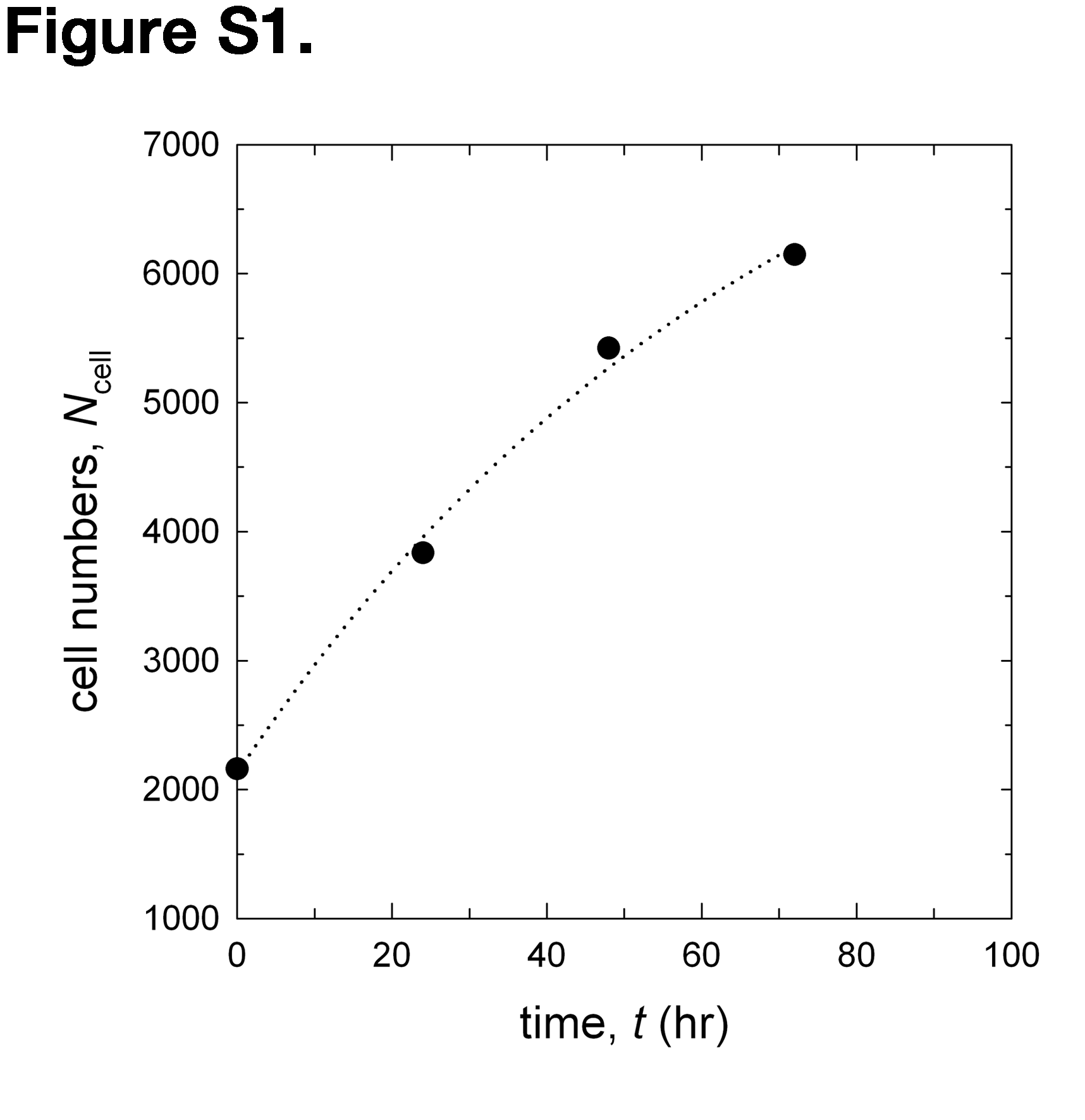

Supplement: Figure S1 — Doubling time of uninduced EOH cells. We employed regression with “exponential rise to maximum” type, as y = yo + b(1 – e–ax), where yo is the intercept, a is the slope between x and y, and b is the maximum y value. According to the estimations in 99% confidence (P < 0.01), Ncell at t = 0 corresponds to 2128 and the growth rate (i.e., number of doublings that occur per unit of time) results in 0.0139. Note that our data can be well fitted as Ncell = 2128+6469 (1 – e−0.0139×time). The doubling time of EOH cells is calculated as ln2/0.0139 = 49.87 hr. (TIF) [file pone.0035244.s001.tif]
